# *pygid*: a Python package for fast data reduction in grazing-incidence diffraction

**DOI:** 10.1107/S1600576725010593

**Published:** 2026-02-01

**Authors:** Ainur Abukaev, Constantin Völter, Mikhail Romodin, Sebastian Schwartzkopff, Florian Bertram, Oleg Konovalov, Alexander Hinderhofer, Dmitry Lapkin, Frank Schreiber

**Affiliations:** a Institut für Angewandte Physik, Universität Tübingen, Auf der Morgenstelle 10, 72076 Tübingen, Germany; b Deutsches Elektronen-Synchrotron DESY Notkestraße 85 22607 Hamburg Germany; c European Synchrotron Radiation Facility (ESRF), 71 avenue des Martyrs, 38000 Grenoble, France; DESY, Hamburg, Germany

**Keywords:** Python packages, data reduction, data analysis, grazing-incidence X-ray diffraction GIXD, grazing-incidence wide-angle X-ray scattering GIWAXS, grazing-incidence small-angle X-ray scattering GISAXS, wide-angle X-ray scattering WAXS, small-angle X-ray scattering SAXS

## Abstract

Advances in X-ray and neutron sources, as well as in area-detector technologies, enable the recording of several terabytes of raw two-dimensional detector data in a single experiment. While several efficient integration and conversion tools are available for data collected in transmission geometry, analogous solutions for grazing-incidence diffraction (including grazing-incidence X-ray diffraction and grazing-incidence wide-angle X-ray scattering) experiments have not yet achieved the same level of efficiency. The development of new data analysis tools, including machine-learning-based software for X-ray data, necessitates the establishment of a standardized format for the converted data. To address these challenges, we have developed a new Python library, *pygid*, which is designed to facilitate fast data processing while providing compatibility with various raw data formats, a standardized data storage format and an intuitive interface for straightforward use. *pygid* supports three types of coordinate systems and both transmission and grazing-incidence geometries. It is capable of handling large datasets, performing one-dimensional line cuts and simulating expected Bragg peak positions for given structures. The package facilitates sample and experimental metadata curation in accordance with the FAIR principles. As an integral part of the broader *mlgid* pipeline, *pygid* serves as the initial step linking raw scattering patterns with machine learning tools for data analysis. The *pygid* package is accessible at https://github.com/mlgid-project.

## Abstract 2

A new Python package, *pygid*, enables fast batch processing of X-ray scattering data, with a particular focus on grazing-incidence geometries.

## 1. Introduction

X-ray and neutron scattering techniques are essential tools in materials science, chemistry, biophysics and condensed matter physics. Their widespread use is supported by the continuous progress in large-scale X-ray and neutron source infrastructures, which provide high-brilliance and tunable radiation for advanced structural investigations (Willmott, 2019 ▸). At the same time, the development of modern 2D detectors with continuous readout and minimal dead times down to 100 ns has significantly improved spatial and temporal resolution in scattering measurements, enabling fast data acquisition in *e.g.* time-resolved and *in situ* experiments (Bein *et al.*, 2015 ▸; Bommel *et al.*, 2014 ▸; Eres *et al.*, 2019 ▸; Ferrer *et al.*, 2013 ▸; Ju *et al.*, 2021 ▸; Kowarik *et al.*, 2006 ▸; Magnussen *et al.*, 2024 ▸; Nicklin *et al.*, 2017 ▸; Richard *et al.*, 2010 ▸; Ulbrandt *et al.*, 2020 ▸; Zhang *et al.*, 2024 ▸). As a result, the growing volume and complexity of collected data have created a need for efficient and scalable software tools capable of reliable data reduction and analysis.

Among the various X-ray scattering techniques, grazing-incidence wide- and small-angle X-ray scattering (GIWAXS/GISAXS) methods have become indispensable tools for studying thin films, nanostructured materials and surfaces (Banerjee *et al.*, 2021 ▸; Eisenberger & Marra, 1981 ▸; Feidenhans’l, 1989 ▸; Smilgies, 2025 ▸; Werzer *et al.*, 2024 ▸). These methods utilize an incident X-ray beam at a shallow angle, around the critical angle of total reflection, maximizing surface sensitivity (Robinson & Tweet, 1992 ▸). GIWAXS provides detailed information on crystal unit cells, atomic/molecular arrangement, degree of crystallinity and orientation of the crystallites on the substrate surface. These techniques have become widely applied in a broad range of research areas, such as organic and hybrid electronics and photovoltaics, where the precise structural characterization of thin films is crucial. Examples include metal halide perovskites (Barrit *et al.*, 2022 ▸; Mundt & Schelhas, 2020 ▸; Schlipf & Müller-Buschbaum, 2017 ▸; Steele *et al.*, 2023 ▸), organic small molecules (Diao *et al.*, 2014 ▸; Gu *et al.*, 2018 ▸; Hodas *et al.*, 2018 ▸; Lapkin *et al.*, 2025 ▸; Richter *et al.*, 2017 ▸; Arias *et al.*, 2021 ▸) and polymers (Manley *et al.*, 2017 ▸; Müller-Buschbaum, 2014 ▸; Posselt *et al.*, 2017 ▸; Yang *et al.*, 2020 ▸). Historically, the term GIXD (grazing-incidence X-ray diffraction) was more commonly used, but it is considered equivalent in this context. GISAXS is particularly suited for characterizing nano- and microscale morphology, including particle distribution, shape and surface roughness, by analyzing intensity scattered at small angles (Kaune *et al.*, 2009 ▸; Smilgies *et al.*, 2002 ▸; Smilgies, 2022 ▸; Smilgies, 2025 ▸). Meanwhile, GISANS – the neutron analog of GISAXS – offers complementary advantages for soft and organic materials due to its sensitivity to light elements like hydrogen, while isotopic substitution allows for tunable contrast variation (Dosch, 1992 ▸; Hamilton *et al.*, 1994 ▸; Jones *et al.*, 1999 ▸; Müller-Buschbaum *et al.*, 2003 ▸; Müller-Buschbaum, 2013 ▸; Steitz *et al.*, 2004 ▸). However, the complexity of the grazing-incidence geometry, including symmetry breaking and distortion of peaks, poses challenges for data reduction, intensity correction and analysis compared with transmission scattering experiments.

A fundamental step in grazing-incidence diffraction (GID) data analysis is the conversion of raw 2D detector scattering patterns into physically meaningful cylindrical coordinates (*q*_*xy*_, *q*_*z*_) – the in-plane and out-of-plane components of the scattering vector – or/and into polar coordinates (*q*_abs_, χ) – the absolute value of the scattering vector and azimuthal angle (Section 3.2 and Appendix *A*[App appa]). This process should also be accompanied by various intensity corrections and masking of dead and hot pixels (Section 4). Several software tools have been developed to facilitate GID data processing, mostly written in Python and MATLAB programming languages. *GIXSGUI* is a MATLAB-based tool with script-based access and a graphical user interface (GUI) (Jiang, 2015 ▸). It provides software for 2D data visualization, reduction, line cutting and indexing of grazing-incidence X-ray scattering data, and for handling large datasets, such as those generated in *in situ* and *in operando* studies at synchrotron facilities. *INSIGHT* (*in situ* GIXS heuristic tool) is an object-oriented Python package that can work with data batches (Reus *et al.*, 2024 ▸). The main feature of *INSIGHT* is the usage of frame-to-frame corrections of experimental parameters, such as sample-to-detector distance, that can be changed due to thermal expansions during *in situ* experiments. *GIWAXS-SIIRkit* is a MATLAB-based package designed for quantitative structural characterization of thin films using GIWAXS scattering patterns (Savikhin *et al.*, 2020 ▸). One of its key features is the ability to assess scattering intensity variations by considering factors such as refractive index shift and incident beam footprint. *indexGIXS* provides a GUI for experimental data visualization, scattering pattern simulation and peak indexing (Smilgies & Li, 2021 ▸). *pyFAI* (Python fast azimuthal integration) is optimized for fast data reduction, supporting azimuthal integration and detector calibration. The package provides a pixel-splitting method for conversion and offers the fastest 1D integration time (down to 48 ms for a 4 megapixel pattern on a four-core office computer) (Ashiotis *et al.*, 2015 ▸). The present work introduces the new Python-based package *pygid*. Our approach considers both the functionality and practical experience gained from existing packages. *pygid* features increased efficiency and an extended range of intensity corrections.

As experimental techniques advance and high-throughput measurements become more common, traditional analysis approaches struggle to keep pace with the sheer amount of information collected. In this context, machine learning (ML) has received growing attention and development in the past few years. It has emerged as a powerful tool for large and complex datasets (Starostin *et al.*, 2022*a* ▸; Starostin *et al.*, 2022*b* ▸; Pithan *et al.*, 2023 ▸; Völter *et al.*, 2025 ▸; Ziletti *et al.*, 2018 ▸). However, to exploit the potential of this approach fully, a standardized data format, which *pygid* provides, is essential to facilitate seamless integration with analysis software.

This article details the architecture, geometry conventions and data processing workflow of *pygid* and provides usage examples for different experimental setups. The article is part of a series of papers (Starostin *et al.*, 2022*a* ▸; Starostin *et al.*, 2022*b* ▸; Völter *et al.*, 2025 ▸) focused on GID data acquisition and analysis, with *pygid* serving as the first component of our data processing pipeline *mlgid* that bridges raw detector output and structural characterization.

## 2. The *pygid* package

The package was developed for fast GID data reduction, including grazing-incidence small- and wide-angle scattering experiments using both X-rays and neutrons (GISAXS, GIWAXS, GISANS). It supports a wide range of raw scattering pattern formats, performs 1D and 2D data conversions, and saves data in a standardized format. The ability to process batches of raw data makes it suitable for integration into synchrotron and neutron beamlines for online data reduction during measurements. The simple and intuitive design, along with the examples and documentation provided, makes it user friendly.

Python was chosen as the programming language for the development of the *pygid* package due to its readability, flexibility and extensive ecosystem of libraries, which make it particularly well suited for data analysis and rapid prototyping (Nagpal & Gabrani, 2019 ▸; Saabith *et al.*, 2019 ▸). Additionally, Python’s versatility and the modular nature of the *pygid* package allow for easy integration with other libraries and software and with scientific workflows, enabling seamless interaction with existing tools and systems, including synchrotron beamlines. However, since Python is an interpreted language it lacks computational performance when processing large datasets. To compensate for that, we used the *numexpr* library, which minimizes memory access and significantly accelerates mathematical operations by utilizing optimized multi-threading (McLeod *et al.*, 2018 ▸). For data conversion, we implemented the *OpenCV-Python* package (Bradski, 2000 ▸), which efficiently handles image processing tasks, enabling fast conversion and manipulation of 2D detector data. This combination of the Python environment for flexibility and the C-based libraries *numexpr* and *OpenCV* for computational speed allowed us to create an effective and high-performance tool for working with X-ray diffraction data, providing a balance between usability and performance.

## 3. Data processing flow

**Figure 1:**
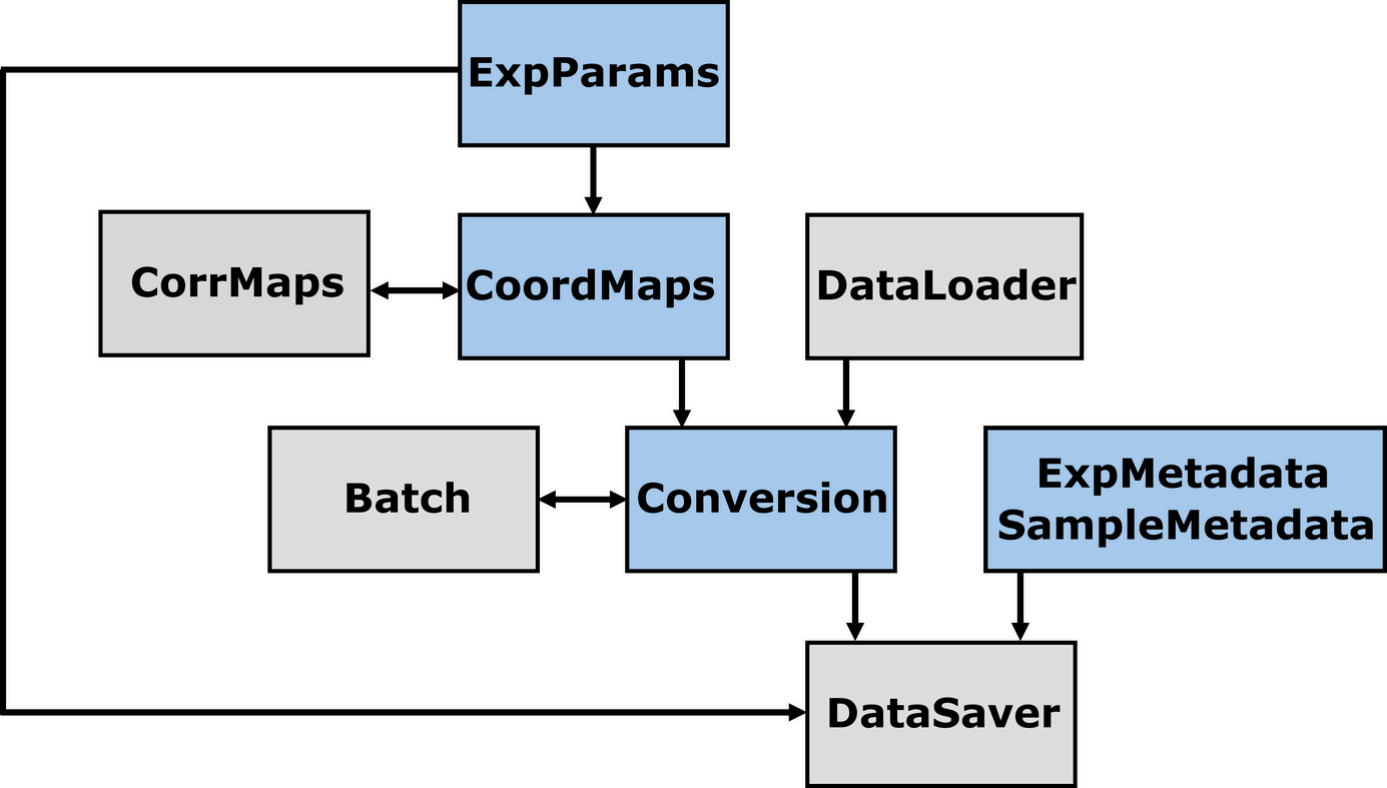
Conceptual architecture of *pygid*. Blue boxes correspond to the classes that the user interacts with, while gray boxes represent internal classes.

In this section, we describe the structure of the *pygid* package and the processing pipeline for raw data within the script, including geometry representation, experimental data handling and metadata curation. The first step of the conceptual design of *pygid* involves calculating coordinate maps and intensity correction matrices based on the experimental parameters (Fig. 1 ▸). Raw data loaded from the specified path are intensity-corrected, masked and then transformed using these maps. To facilitate the handling of large datasets, batch processing can be enabled. Finally, the processed data in 32-bit floating-point format, along with sample metadata and experimental parameters, are stored for further analysis.

### 3.1. Experimental parameters

To store and operate the experimental parameters, *pygid* uses a class named ExpParams. It defines six parameters related to the detector orientation: the sample position projection onto the detector plane (poni1, poni2 in metres) or the direct beam position (centerX, centerY in pixels), the sample-to-detector distance (SDD) along the normal to the detector plane before applying any rotations, and three detector rotation angles around the laboratory coordinate axes (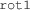, 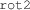, 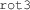) with the origin at the sample positions. Additionally, it stores experimental details such as the X-ray wavelength, detector pixel size, and image transformation flags (fliplr, flipud, transp) for horizontal flipping, vertical flipping and transpose, respectively. All these parameters, except for the last set of keys, can be imported from a PONI file created using the *pyFAI* package or its GUI (Ashiotis *et al.*, 2015 ▸). However, manual input of these values is also supported. The ExpParams class can additionally handle both static and dynamic masks. Users can provide either a 2D array for the static mask (mask) or a file path (mask_path) pointing to a mask file in *NumPy* (https://numpy.org/), EDF or TIFF format. The static mask is applied uniformly to all images to exclude detector gaps, the direct beam region or the beam-stop shadow. Dynamic masks, in contrast, are generated from each raw scattering frame and are based on user-defined minimum and maximum intensity thresholds (count_range), effectively excluding hot and dead pixels.

### 3.2. Coordinate map calculation

The functionality of the CoordMaps class can be described in three steps:

(i) computation of the detector pixel coordinates in Cartesian (*q*_1_, *q*_2_), cylindrical (*q*_*xy*_, *q*_*z*_), polar (*q*_abs_, χ) or pseudo-polar (*q*_abs_, *q*_abs_χ) systems in reciprocal space for both transmission and GID geometries;

(ii) estimation of maximum measured *q* values based on the detector position and size (optional);

(iii) calculation of intensity correction matrices (optional).

**Figure 2:**
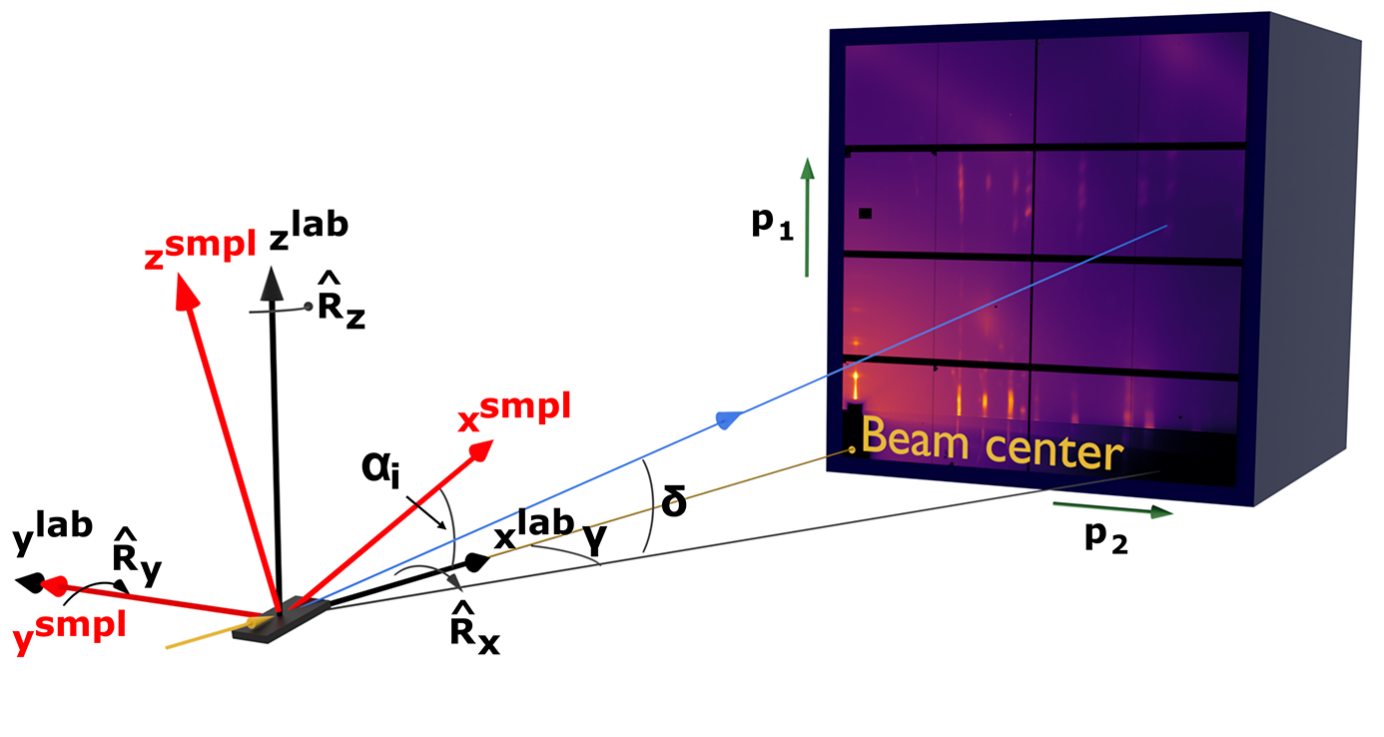
Scattering geometry and the three coordinate systems used in *pygid*: (*p*_1_, *p*_2_) – pixel positions in the detector real-space coordinate system (green); (*x*^lab^, *y*^lab^, *z*^lab^) – laboratory reciprocal-space coordinate system used in transmission geometry (black); (*x*^smpl^, *y*^smpl^, *z*^smpl^) – sample coordinate system in reciprocal space, rotated by the angle of incidence α_i_ counter-clockwise around the *y*_lab_ axis, for grazing-incidence geometry (red), direct beam (yellow) and scattered beam (blue) with horizontal (γ) and vertical (δ) scattering angles. The angle of incidence α_i_ is exaggerated for clarity in the visualization.

All calculations rely on three mutually connected orthonormal right-handed coordinate systems (Fig. 2 ▸), similar to those described by Breiby *et al.* (2008 ▸) and Smilgies & Blasini (2007 ▸). The first is the detector coordinate system (DCS) in real space (*d*_1_, *d*_2_, *d*_3_), which is defined by pixel positions (*p*_1_, *p*_2_) in the raw pattern and the SDD. The second is the laboratory coordinate system (LCS) in reciprocal space (indicated by the superscript lab, *e.g.q*^lab^), centered at the point where the X-ray beam intersects the sample. In this system, the direct beam propagates along the *x*^lab^ axis. These two frames are related through detector rotations (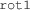, 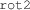, 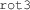) around the laboratory coordinate frame. While this description is sufficient for transmission geometry, grazing-incidence experiments require an additional sample coordinate system (SCS, denoted using the superscript smpl). This system is linked to the laboratory frame via a rotation matrix around the *y*^lab^ axis to the angle of incidence.

**Figure 3:**
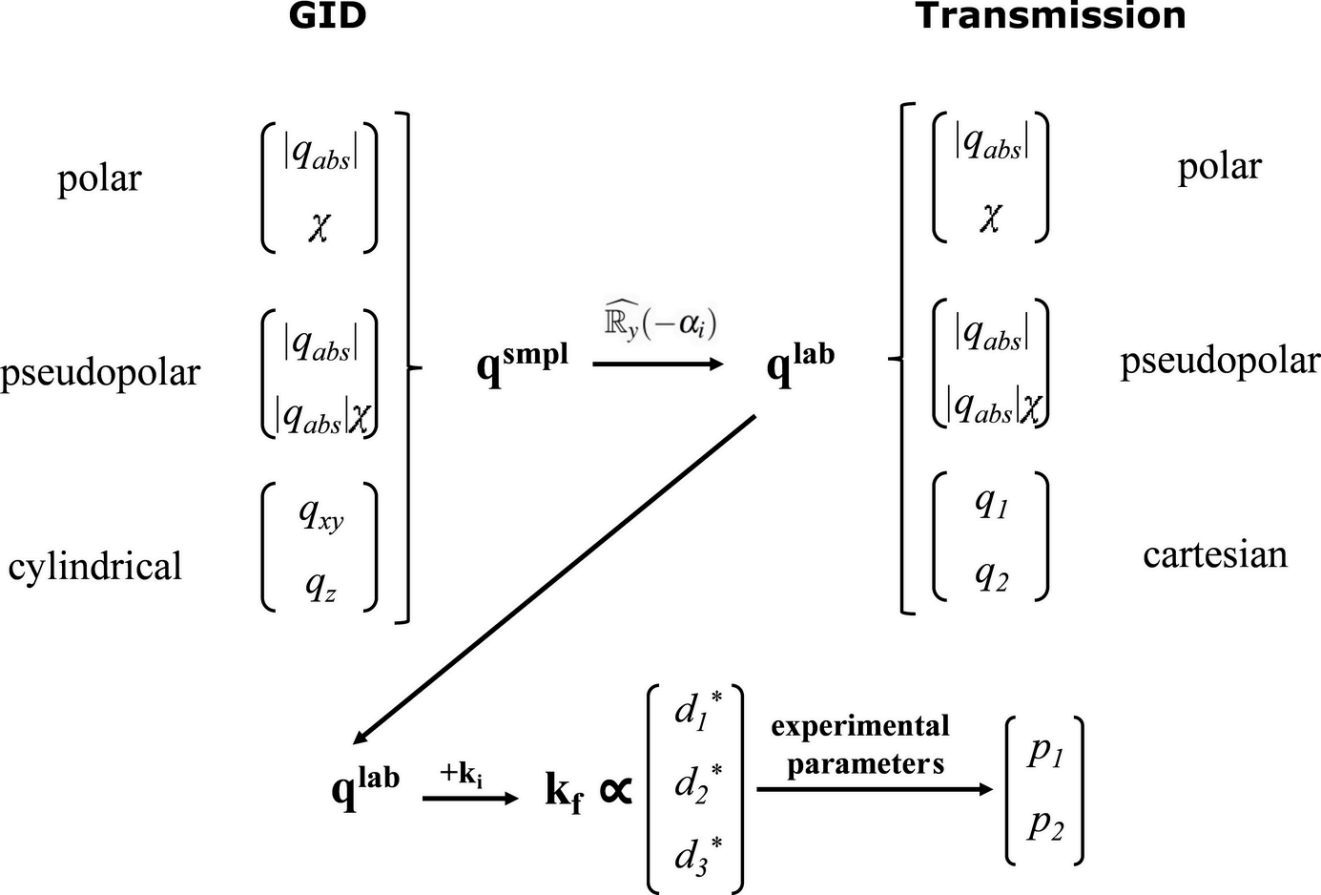
Schematic representation of coordinate map calculation from the given coordinate types and ranges. **q**^smpl^ and **q**^lab^ are scattering vectors in the sample and laboratory coordinate systems, respectively, related to each other via a rotation matrix around the *y*^lab^ axis. **k**_i_ is the incident wavevector in the LCS, **k**_f_ the scattered wavevector in the LCS, (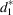, 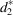, 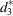) the real-space pixel positions in the LCS and (*p*_1_, *p*_2_) the pixel positions in the converted images.

The primary function of the CoordMaps class is to compute pixel coordinates of the transformed image in detector space, given predefined coordinate ranges (Fig. 3 ▸). First, the given ranges in polar cylindrical (*q*_*xy*_, *q*_*z*_), (*q*_abs_, χ), pseudo-polar (*q*_abs_, *q*_abs_χ) or 2D Cartesian (*q*_1_, *q*_2_) coordinates are transformed into Cartesian *q*-space coordinates, **q** = (*q*_*x*_, *q*_*y*_, *q*_*z*_). In the case of GID geometry, the calculated **q** vector is initially defined in the sample coordinate system and is then rotated by the incidence angle to be represented in the laboratory coordinate system. The corresponding final wavevector **k**_f_ is then calculated in the LCS as **k**_f_ = **k**_i_ + **q**, where **k**_i_ = (2π/λ, 0, 0). To transform **k**_f_ into detector space, three rotation matrices, defined by the detector rotation angles (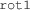, 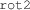, 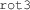), are applied. The resulting vector is proportional to the pixel position 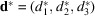 in real space. In the final step, the pixel coordinates (*p*_1_, *p*_2_) are computed from 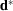 using experimental parameters, including the direct beam position and pixel size.

A key feature of the *pygid* package is that it reuses the calculated coordinate map (*p*_1_, *p*_2_), representing the converted image pixel positions, multiple times for different scattering patterns recorded under the same experimental conditions (*e.g.* fixed angle of incidence for time scans). This reusability significantly reduces the conversion time, as the coordinate map does not need to be recalculated for each individual pattern, thereby improving the overall efficiency of the data processing pipeline.

The estimation of the *q* range is based on the opposite conversion process from the pixel coordinates of the raw image in detector space to *q* values in laboratory and sample spaces for transmission and GID, respectively. Only corner pixels and edge pixels on the same horizontal and vertical lines as the direct beam pixel are processed for maximum scattering vector and *q* values in cylindrical (*q*_*xy*_, *q*_*z*_) and Cartesian (*q*_1_, *q*_2_) calculations. However, for angular range evaluation all border pixels are processed. Finally, intensity correction matrices require pixel positions in reciprocal space for each pixel of the raw scattering pattern. The implemented intensity corrections will be described further in Section 4.

### 3.3. Data loading

The DataLoading class is designed to handle raw detector image files in a variety of formats. It supports file types that can be opened by the *FabIO* library (EDF, TIFF, CBF) (Knudsen *et al.*, 2013 ▸) and the *H5py* library (Collette *et al.*, 2023 ▸) for files with more complex structure, including HDF5 and NeXus formats. The *FabIO* library provides efficient access to a wide range of 2D detector images. The *H5py* library has demonstrated superior performance in terms of data loading speed compared with other packages such as *PyTables* (Alted *et al.*, 2002 ▸), *netCDF4* (Pierce, 2025 ▸) and *h5netcdf* for HDF5 files (Table S1 in the supporting information). However, the actual loading time is highly dependent on the data storage infrastructure, the internal file structure of the files, and any external references to other libraries or resources.

Users also have the option to load the scattering patterns externally and transfer the raw data as 2D or 3D arrays into *pygid*. The DataLoading class operates internally and is not intended for direct user interaction. Instead, users interact with the Conversion class, where they specify the data file path and the location of raw data arrays (for HDF5 and Nexus files). These arrays are then processed and prepared for subsequent analysis.

### 3.4. Conversion

The preliminary calculated coordinate maps and loaded data are passed to the Conversion class, which first applies the correction matrices calculated in the CoordMaps class. According to the calculated coordinate map, the image can be represented in polar, pseudo-polar, cylindrical or 2D Cartesian coordinates (Table 1 ▸).

The primary remapping function utilizes geometric image transformations from the *OpenCV-Python* library [cv2.remap()] (Gonzalez & Woods, 2018 ▸). Since new pixel positions may be non-integer and pixel intensities need to be accurately estimated, the package employs several inter­polation techniques. Five interpolation methods are implemented in the script: nearest-neighbor, bilinear, bicubic and Lanczos (Cullum & Willoughby, 2002 ▸) interpolation, and resampling based on the pixel area relation. This allows users to balance speed and quality depending on the task, whether it involves image downscaling or upscaling.

### 3.5. Data saving

To store individual converted images, complete datasets and even multiple datasets within a single file, we employ the widely adopted NeXus format (Klosowski *et al.*, 1997 ▸; Könnecke *et al.*, 2015 ▸), which provides a standardized framework for data exchange and archiving in neutron and X-ray experiments. File writing is implemented using the *H5py* library (Collette *et al.*, 2023 ▸). The format allows for the storage not only of converted patterns but also of experimental parameters and sample descriptions.

**Figure 4:**
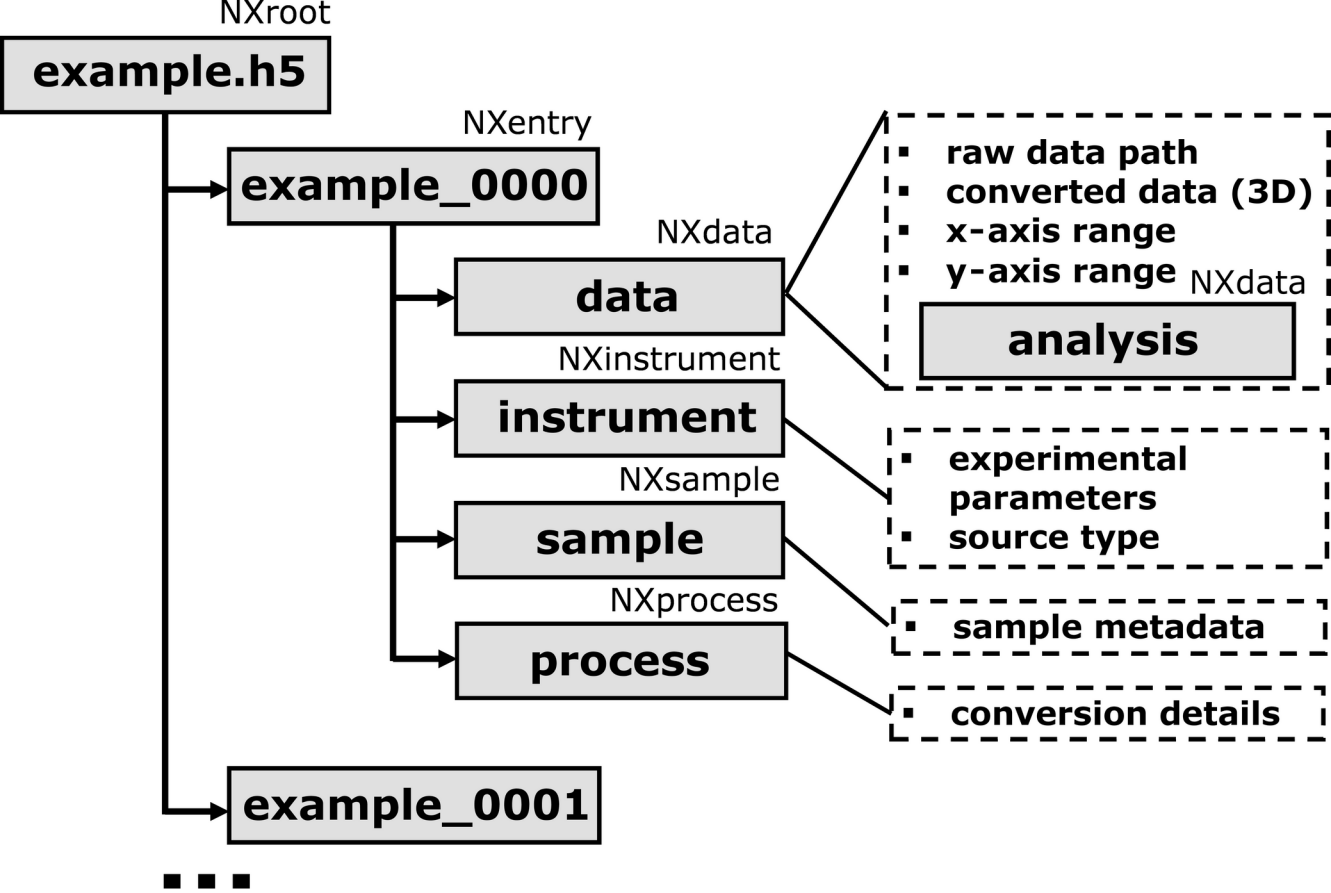
Overview of the structure of a saved NeXus file as a modified NXsas application definition.

The data type closely related to GIWAXS/GISAXS data is the NXsas application definition, which was designed for storing small-angle scattering (SAS) data in the NeXus format. However, we have slightly modified the data group to store arrays of scattering data for motor or time scans, as is implemented in the NXscan definition (Fig. 4 ▸). An additional analysis group is used to store the results of peak detection and fitting at the next analysis step. Converted images can be stacked to the previously calculated data arrays if they have the same shape, or can be saved in a separate NXentry group. The naming of datasets for different types of coordinates is shown in Tables 1 and 2. Additionally, a single converted image can be visualized and saved using the *matplotlib* library, which supports both vector (PDF, SVG, EPS, PGF) and raster (PNG, JPG/JPEG, TIFF, BMP) formats (Hunter, 2007 ▸).

The instrument group contains data from the ExpParams class, following the standard naming defined in the NXsas format. Additional information about the experiment and source can be added using the ExpMetadata class (Table S2). Details of the transformation, such as the date and the applied intensity corrections, are stored in the process group.

Finally, the sample group stores the sample-related metadata. We strongly recommend that the metadata include the sample name, structure, preparation description and experimental conditions via the SampleMetadata class (Tables S2 and S3). Sample metadata can also be imported directly from a YAML file similar to the ORSO (Open Reflectometry Standards Organization, https://www.reflectometry.org/) specification. Users may further extend the sample group with custom fields, for example chemical formula, temperature, pressure, mass *etc.*, as proposed by the DAPHNE4NFDI initiative (Barty *et al.*, 2023 ▸; Lohstroh *et al.*, 2024 ▸; Amelung *et al.*, 2025 ▸) in accordance with the FAIR Guiding Principles (Wilkinson *et al.*, 2016 ▸). Both metadata classes support formats such as strings, lists, integer or float values, and *NumPy* arrays.

### 3.6. Other features

#### 3.6.1. Batch analysis

The package automatically uses the batch mode when the number of loaded patterns is larger than batch_size (32 is the default value). Here, scattering patterns are loaded in batches when the remapping function is called. After remapping, the loaded scattering patterns are deleted from the memory to allow the next batch to be processed and to avoid extensive memory usage. The result will not be plotted and returned; only saving in an HDF5 file is allowed.

#### 3.6.2. Line profiles

In addition to 2D conversion functions, we have added 1D radial and azimuthal integration functions (Table 2 ▸). The corresponding functions call remapping into polar coordinates in sample or laboratory spaces and perform averaging along the angular and radial axis. Users can adjust the resolution in both angular and radial directions, depending on their preference for accuracy or speed. For GID data, it is also possible to obtain horizontal profiles that are calculated from patterns in cylindrical coordinates by averaging in a small *q*_*z*_ range close to 0. For vertical profiles, which are not directly accessible due to the missing edge, we recommend calculating radial profiles with a small angular range close to the vertical axis according to the size of the missing wedge. The profiles can be plotted with adjustable color map, limits and distance between curves for multiple datasets, saved as a figure or in an HDF5 file, and returned as a *NumPy* array.

#### 3.6.3. GIWAXS pattern simulation

We integrated the *pygidSIM* package (Romodin, 2025 ▸), which simulates expected Bragg peak positions in GIWAXS patterns based on crystallographic information (unit-cell parameters and atomic positions) provided in CIFs (Hall *et al.*, 1991 ▸). The package outputs the positions as cylindrical coordinates and intensities of Bragg reflections for various Miller indices. *pygid* enables users to overlay experimental patterns with simulated data for a set of CIFs and crystal orientations.

## 4. Intensity corrections

The importance and details of intensity corrections for 2D detector data in X-ray scattering experiments have been discussed in several articles (Gasser *et al.*, 2025 ▸; Jiang, 2015 ▸; Pauw *et al.*, 2017 ▸). While the positions of Bragg peaks in WAXS/GIWAXS experiments provide information about the crystal type and unit-cell parameters, their intensities make it possible to estimate the amplitude of structure factors corresponding to the arrangement of individual atoms within the unit cell. In SAXS/GISAXS experiments, the intensity of the scattering pattern contributes to the scattering-invariant parameters of phase separation efficiency and intermolecular structure dispersity. This underscores the importance of accurate corrections, even in small-angle scattering, despite the reduced impact of most corrections due to the large sample-to-detector distances. Since many of these corrections are independent of the angle of incidence, *pygid* applies them to the raw scattering pattern prior to conversion. A list of the *pygid* corrections, along with their input parameters and types, is presented in Table 3 ▸.

(i) Flat-field correction accounts for the varying sensitivity of different detector pixels. The creation of the corresponding correction matrix is not a part of the *pygid* workflow but can be performed *e.g.* using the approach described by Weng *et al.* (2023 ▸). The matrix is then loaded as a 2D array.

(ii) Dark-current correction involves subtracting the signal recorded in the absence of the X-ray beam. The correction matrix should be loaded as a 2D array.

(iii) Solid-angle correction accounts for the geometric effects arising from the proportional relationship between the signal detected by a pixel and its corresponding solid angle ΔΩ,
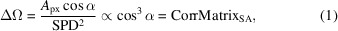
where *A*_px_ is the pixel area, SPD is the sample-to-pixel distance, and α is the angle between the normal vector to the pixel surface **n** and the wavevector **k**_f_ corresponding to that pixel position.

(iv) Polarization correction accounts for the polarization of the incident X-ray beam. The scattered intensity depends on the angle between the polarization of the incident and scattered waves, following a squared cosine dependence. This can be described by the horizontal (γ) and vertical (δ) scattering angles in both in-plane and out-of-plane scattering geometries,
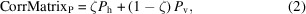
where the subscripts h and v refer to, respectively, horizontal and vertical components and 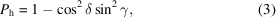

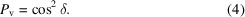
The polarization parameter ζ is approximately equal to 1 for typical synchrotron radiation (horizontal polarization) and 0.5 for an unpolarized laboratory X-ray tube. Users are required to specify the ζ parameter using the pol_type key.

(v) Air attenuation correction is based on the Beer–Lambert extinction law and arises due to the varying X-ray beam paths to each pixel. The linear attenuation coefficient (μ_air_) depends on the X-ray energy and air density and must be provided by the user, while the sample-to-pixel distance (SPD) is derived from the coordinate maps: 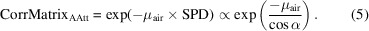
(vi) Sensor attenuation and sample absorption corrections are based on the X-ray beam path through the detector sensor and sample. Linear attenuation coefficients and thicknesses are required for these calculations. For a more detailed description of the correction process and its mathematical aspects, we refer the reader to Gasser *et al.* (2025 ▸).

(vii) The Lorentz correction is related to the distribution of the Bragg peaks on circles/spheres with different radii in reciprocal space (Jiang, 2015 ▸). The usage of this correction in *pygid* is limited to the most common thin-film 2D and 3D powder-like cases, which can be chosen using powder_dim = 2 or 3, respectively:
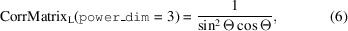

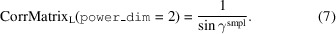
Here γ^smpl^ is the horizontal scattering angle and 2Θ is the total scattering angle in the SCS.

## 5. Performance

To assess the performance of the *pygid* package, we conducted benchmark tests on three computing systems with different capabilities: a typical office desktop PC (Intel i5-6500, four cores, 16 GB RAM, Windows 10), the ESRF VISA cluster (AMD CPU, 32 cores, 128 GB RAM; https://visa.readthedocs.io/en/latest/) and the DESY Maxwell cluster (AMD CPU, 48 cores, 512 GB RAM; https://docs.desy.de/maxwell/). As a test dataset, we used an HDF5 file with a single GIWAXS pattern of diindenoperylene (DIP), acquired with an EIGER2 X CdTe 4M detector (2162 × 2068 pixels) on the ID10-SURF beamline at the ESRF. The X-ray energy was *E* = 20 keV. To test the multiprocessing mode (MP) during conversion and coordinate map calculations, we used an HDF5 file containing 13 scattering patterns measured at different angles of incidence (ranging from 0.04° to 0.1°) using the same experimental setup. For each angle, a separate coordinate map was calculated in MP mode. Preliminary calibration was based on the lanthanum hexaboride (LaB_6_) scattering pattern using the *pyFAI* GUI (Ashiotis *et al.*, 2015 ▸). Each performance test was averaged over 100 runs.

Table 4 ▸ summarizes the execution times for different stages of the *pygid* workflow. The initial stage of preliminary calculations, which includes pixel position transformation into cylindrical coordinates, generation of polarization and solid-angle correction maps, and determination of converted axis ranges, takes approximately 1161 ± 32 ms on an office PC. This step is slightly faster on the VISA and Maxwell clusters, requiring 1001 ± 122 and 689 ± 3 ms, respectively. Here, the calculated *q*_*xy*_ and *q*_*z*_ ranges were [0, 4.32] and [0, 4.06], respectively. The resolution was determined automatically on the basis of the pixel size. As a result, the converted image had a size of 1749 × 1861 pixels. The second stage, performed for each scattering pattern, involves raw data loading, conversion with linear pixel interpolation and saving into an HDF5 file, taking 103 ± 11 and 105 ± 25 ms on the office PC and Maxwell cluster, respectively, while the VISA cluster reduced this processing time to 84 ± 4 ms. As the file input/output performance may vary depending on the storage system and specific configuration, the following analysis focuses on the core image conversion functionality and the coordinate map computation, which represents the most computationally demanding step.

Thus, the execution time of the coordinate map calculation scales nearly linearly with the resolution, *i.e.* the size of the converted image (Fig. S1 and Table 4 ▸). The highest calculation speed was observed on the Maxwell cluster. When multiple coordinate maps were calculated in multiprocessing mode, the processing speed increased significantly, by up to 45%, 83% and 81% for the PC and the VISA and Maxwell clusters, respectively.

**Figure 5:**
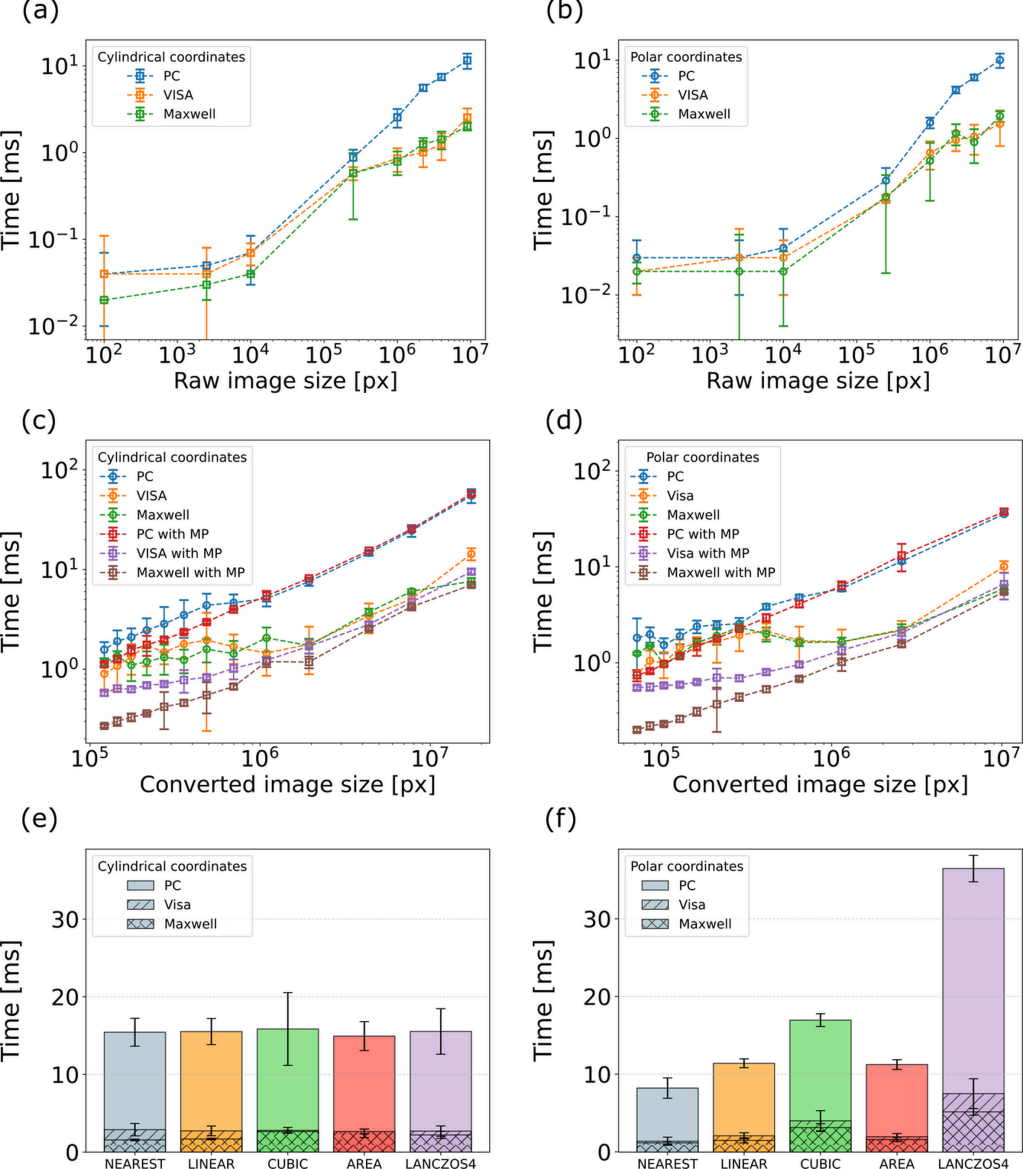
Performance results of the *pygid* package, showing the dependence of the conversion time on (*a*) and (*b*) raw pattern size, (*c*) and (*d*) resolution, and (*e*) and (*f*) interpolation type for (*a*), (*c*) and (*e*) cylindrical and (*b*), (*d*) and (*f*) polar coordinates.

The conversion time depends on both the raw scattering pattern size [Figs. 5 ▸(*a*) and 5 ▸(*b*)] and the chosen resolution [Figs. 5 ▸(*c*) and 5 ▸(*d*)]. The benefits of multiprocessing become apparent primarily on cluster systems: the conversion time was reduced by up to 34% on the VISA cluster and up to 43% on the Maxwell cluster.

As previously mentioned, the conversion speed can also be influenced by the interpolation method [Figs. 5 ▸(*e*) and 5 ▸(*f*)]. For conversion to cylindrical coordinates no significant differences were observed. On the other hand, for polar coordinates, nearest-neighbor interpolation resulted in faster processing, whereas more complex methods, such as cubic and Lanczos (Cullum & Willoughby, 2002 ▸), significantly slowed down the conversion. The differences between interpolation methods are illustrated in Fig. S2. In our previous work, we demonstrated that the interpolation can introduce artifacts in the low-*q* region, which consequently affect the ML-based peak detection process (Völter *et al.*, 2025 ▸). From this perspective, the pixel-splitting approach implemented in the *pyFAI* package is more suitable. However, interpolation remains crucial in the high-*q* region and near the missing wedge, where some bins may be empty after conversion. These differences may become more pronounced for lower-resolution images. Therefore, users are encouraged to choose the interpolation method that best suits their specific case, considering resolution, processing time and the region of interest.

## 6. Data reduction and simulation examples

**Figure 6:**
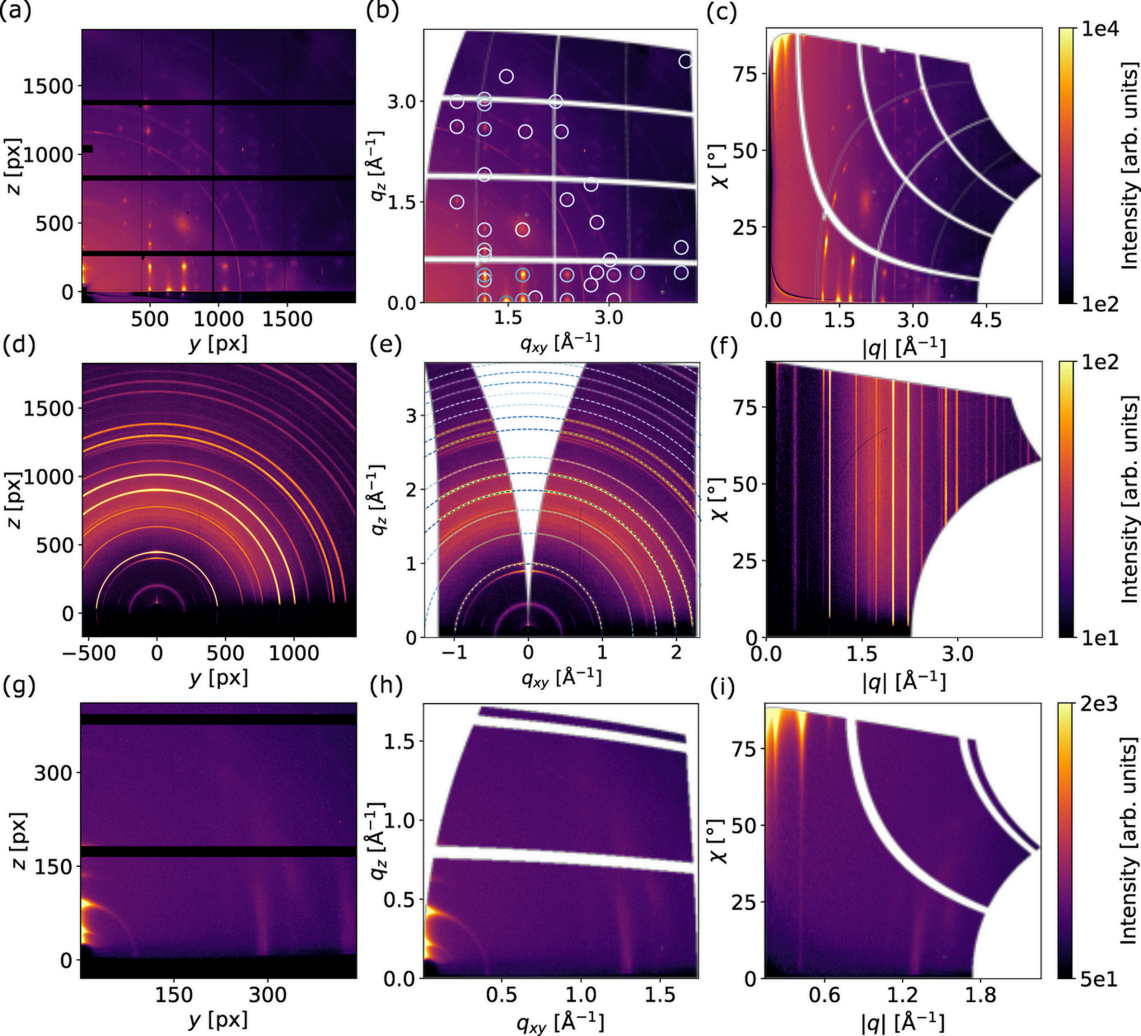
*pygid* package usage examples. (*a*)–(*c*) GIWAXS patterns of a DIP thin film measured on the ESRF ID10 beamline (EIGER2 X CdTe 4M detector). (*d*)–(*f*) GIWAXS patterns of MAPbI_3_ measured on the DESY P08 beamline (PerkinElmer flat-panel detector). (*g*)–(*i*) GIWAXS patterns of Pb nanoparticles measured using a laboratory scattering setup (Pilatus 300k detector). For each dataset, (*a*), (*d*), (*g*) raw scattering patterns, (*b*), (*e*), (*h*) converted images in cylindrical coordinates and (*c*), (*f*), (*i*) converted images in polar coordinates. The simulation of the scattering patterns was done using the *pygidSIM* package (blue rings). The color of the simulated data is proportional to the structure factor.

As a demonstration of the capabilities of the *pygid* package, we present three examples of data obtained using different detectors at various X-ray sources, each with distinct raw data formats. Fig. 6 ▸ shows the raw scattering patterns alongside their representations in cylindrical and polar coordinates after transformation.

The first example features the GIWAXS pattern of a DIP thin film acquired on the ESRF ID10 beamline (X-ray beam energy *E* = 20 keV). The scattering data were recorded using an EIGER2 X CdTe 4M detector (2162 × 2068 pixels) and saved in the NeXus format. A Python-based script for coordinate transformation and expected peak position simulation is provided in Table 5 ▸ as an example of *pygid* package usage. The resulting pattern reveals a highly oriented 2D DIP powder structure [Figs. 6 ▸(*a*) to 6 ▸(*c*)]. For simulations using *pygidSIM*, a monoclinic σ-phase oriented along the [001] direction was used (Heinrich *et al.*, 2007 ▸). Only peaks with intensities exceeding 0.1% of the maximum are shown in the figure.

Secondly, a typical GIWAXS pattern of methylammonium perovskite MAPbI_3_ is shown (Kneschaurek *et al.*, 2023 ▸). The experiment was conducted on the PETRA III P08 beamline using a PerkinElmer flat-panel detector (2048 × 2048 pixels). The X-ray energy was *E* = 18 keV. The raw data were saved as TIFF format. The tetragonal perovskite structure appears to be randomly oriented [Figs. 6 ▸(*d*) to 6 ▸(*f*)] (Druzbicki *et al.*, 2023 ▸). The rings and arcs observed at 0.45, 0.51 and 0.65 Å^−1^ are not simulated, as they originate from a Pb_3_I_8_ intermediate complex.

Finally, clusters of PbTe nanoplatelets were measured in GID geometry using an in-house X-ray scattering setup (Xeuss 2.0, Xenocs, X-ray beam energy *E* = 8 keV) equipped with a Pilatus 300k detector (487 × 619 pixels). The experiment produced raw data in EDF format. Bragg reflections in the horizontal and vertical directions are observed, indicating the formation of a superlattice from stacked nanoplatelets (Biesterfeld *et al.*, 2024 ▸) [Figs. 6 ▸(*g*) to 6 ▸(*i*)].

The presented examples highlight the usefulness of *pygid* in handling diverse file formats generated using different experimental setups and detectors, including a flat-panel PerkinElmer detector. The integration of the *pygidSIM* package provides a user-friendly framework for simulating the positions and intensities of Bragg reflections and diffraction rings from CIFs.

## 7. Summary and conclusions

The present work introduces the new *pygid* package, developed to address the increasing demand for fast and reliable data reduction of 2D scattering data. As the volume of X-ray and neutron scattering data continues to grow – particularly at high-throughput synchrotron and laboratory facilities – there is a critical need for automated and efficient processing tools. *pygid* is specifically designed for batch conversion and simplifies this process for users with varying levels of expertise. It supports raw data loading across all common formats used in both laboratory and synchrotron environments.

The package is applicable to both small- and wide-angle scattering, in grazing-incidence and transmission geometries. It enables conversion to 2D Cartesian, polar and pseudo-polar reciprocal-space coordinates and provides radial and azimuthal integration routines. The average conversion speed (14.71 ± 0.94 ms per image) is superior to that of other existing tools of 2D remapping and can be further reduced by tuning the resolution, selecting appropriate interpolation methods and using cluster computing. To ensure reliable quantitative analysis, a wide range of intensity corrections have been implemented. In addition, the *pygidSIM* package is included to simulate scattering patterns based on crystallographic information, enabling direct comparison with experimental data. As part of our *mlgid* pipeline, the package provides an interface between raw experimental data and machine-learning-based tools for peak detection, labeling and structure determination.

Future developments will focus on GPU acceleration and enhanced multiprocessing to increase performance further. We also plan to integrate *pygid* directly into beamline workflows at synchrotron facilities, enabling *in situ* data conversion immediately after acquisition, which will help provide rapid feedback during measurements.

## Supplementary Material

The supplementary materials contain recommendations for metadata fields, and additional performance testing results. DOI: 10.1107/S1600576725010593/yr5162sup1.pdf

## Figures and Tables

**Table 1:** 2D conversion types and corresponding axes

Experiment geometry	Converted image type	Function name	Resulting image name	Corresponding axis names
GID	Cylindrical	det2q_gid()	img_gid_q	q_xy, q_z
GID	Polar	det2pol_gid()	img_gid_pol	q_gid_pol, ang_gid_pol
GID	Pseudo-polar	det2pseudopol_gid()	img_gid_pseudopol	q_gid_rad, q_gid_azimuth
Transmission	Cartesian	det2q()	img_q	q_1, q_2
Transmission	Polar	det2pol()	img_pol	q_pol, ang_pol
Transmission	Pseudo-polar	det2pseudopol()	img_pseudopol	q_rad, q_azimuth

**Table 2:** 1D profiling and corresponding axes

Function name	Resulting data name	Corresponding axis name	Description
radial_profile_gid()	rad_cut_gid	q_gid_pol	Makes polar remapping and averages in the given angular range for the GID geometry
radial_profile()	rad_cut	q_pol	Makes polar remapping and averages in the given angular range for the transmission geometry
azim_profile_gid()	azim_cut_gid	ang_gid_pol	Makes polar remapping and averages in the given radial range for the GID geometry
azim_profile()	azim_cut	ang_pol	Makes polar remapping and averages in the given radial range for the transmission geometry
horiz_profile_gid()	horiz_cut_gid	q_xy	Makes cylindrical remapping and averages in the given *q*_*z*_ range for the GID geometry

**Table 3:** Intensity corrections implemented in *pygid*

Correction type	Correction usage key (Boolean)	Variables	Data type
Flat field	–	flat_field	ndarray (2D)
Dark current	–	dark_current	ndarray (2D)
Solid angle	make_solid_angle_corr	–	–
Polarization	make_pol_corr	pol_type	Float ∈ (0, 1)
Air attenuation	make_air_attenuation_corr	air_attenuation_coeff	Float > 0 (m^−1^)
Sensor attenuation	make_sensor_attenuation_corr	sensor_attenuation_coeff	Float > 0 (m^−1^)
		sensor_thickness	Float > 0 (m)
Sample absorption	make_absorption_corr	sample_attenuation_coeff	Float > 0 (m^−1^)
		sample_thickness	Float > 0 (m)
Lorentz	make_lorentz_corr	powder_dim	Integer, 2 or 3

**Table 4:** Comparison of processing time distribution (in ms) across different computational stages Raw scattering patterns in HDF5 format with a resolution of 2162 × 2068 pixels were used for testing.

Computational stage	Office PC	ESRF VISA cluster	DESY Maxwell cluster
Coordinate map calculation	530.88 ± 22.89	455.65 ± 58.75	271.41 ± 2.3
Correction map calculation (with MP)	607.20 ± 22.59 (332.34 ± 26.95)	531.73 ± 106.43 (87.09 ± 3.38)	406.98 ± 1.19 (79.61 ± 0.48)
*q*-range determination	22.63 ± 2.74	13.61 ± 3.71	10.79 ± 0.07
Total time for preliminary calculations	1160.7 ± 32.3	1001.0 ± 121.6	689.18 ± 2.56

Raw data loading	40.95 ± 5.09	30.71 ± 3.78	27.44 ± 0.32
Conversion (with MP)	14.71 ± 0.94 (15.48 ± 0.26)	3.43 ± 1.14 (2.81 ± 0.17)	3.73 ± 0.32 (2.55 ± 0.09)
Data saving	47.55 ± 9.74	49.39 ± 2.0	73.71 ± 24.73
Total time per pattern	103.21 ± 11.03	83.53 ± 4.43	104.88 ± 24.73

**Table 5:** Overview of key steps in *pygid* data reduction workflow

Code	Description
>> import pygid	Import of the package
>> params = pygid.ExpParams(poni_path=’LaB6.poni’, mask_path=’mask.npy’, ai=0.075)	Creation of the ExpParams class instance from the loaded PONI file and mask, and input of incident angle
>> matrix = pygid.CoordMaps(params)	Creation of the CoordMaps class instance
>> exp_metadata = pygid.ExpMetadata(start_time="2024-03-17T14:30:00Z", source_type="synchrotron", source_name="ESRF", detector="eiger4m", instrument_name="ID10")	Metadata definition; an example of the sample metadata is shown in Table S3
>> smpl_metadata = pygid.SampleMetadata(path_to_load="DIP_metadata.yaml")	
>> analysis = pygid.Conversion(matrix=matrix, path="DIP.h5", dataset="/measurement/eiger4m")	Creation of the Conversion class instance and a raw scattering pattern loading
>> analysis.det2q_gid(plot_result=True, save_result=True, path_to_save="DIP_result.h5", exp_metadata=exp_metadata, smpl_metadata=smpl_metadata)	Image conversion to GID cylindrical coordinates, plotting and saving the result with metadata
>> analysis.make_simulation(path_to_cif="DIP_structure.cif", orientation=[0, 0, 1], min_int=1e-3, plot_result=True, return_result=True)	Simulation based on CIF and given orientation, plotting with the experimental scattering pattern and returning Miller indices with positions

## Data Availability

The supplementary materials contain recommendations for metadata fields and additional performance testing results. The *pygid* package is open source and freely available on the GitHub page https://github.com/mlgid-project, along with additional tools for simulation, labeling and machine-learning-based analysis of GID data. The examples presented in the article are available at https://doi.org/10.5281/zenodo.17466183. Additional data supporting the findings of this study are available from the corresponding authors upon reasonable request.

## Appendix A Equations used for coordinate map calculations

From cylindrical () to 3D Cartesian () coordinates in the SCS for a given angle of incidence α_i_:

where *k* is the wavevector magnitude.

From 3D Cartesian () to cylindrical () in the SCS:

From polar (, χ) and pseudo-polar (, ) to cylindrical () in the SCS:

From 2D Cartesian () to 3D Cartesian () in the LCS:

From 3D Cartesian () to 2D Cartesian () in the LCS:

From polar (, χ) and pseudo-polar (, ) to 3D Cartesian () to 2D Cartesian () in the LCS:

The relations between the SCS (**q**^smpl^) and LCS (**q**^lab^) are

The relation between the LCS (**q**^lab^) and detector pixel positions (*p*_1_, *p*_2_) is

where ,  is a rotation matrix over *i* axes of the LCS to Θ, and **d** is a pixel position in the DCS that is related to the pixel coordinates via the pixel size *p*_size_ and the sample projection onto the detector plane (poni1, poni2):
