# Supplementary material for: pygid: a Python package for fast data reduction in grazing-incidence diffraction
Source: J Appl Crystallogr. 2026 Feb 1;59(Pt 1):263–75. doi: 10.1107/S1600576725010593 (PMC12871486; doi:10.1107/S1600576725010593)
Supplement: Supplementary file 1 [file j-59-00263-sup1.pdf]

JOURNAL OF APPLIED CRYSTALLOGRAPHY

Volume 59 (2026)

Supporting information for article:

pygid: a Python package for fast data reduction in grazing-incidence diffraction

Ainur Abukaev, Constantin Völter, Mikhail Romodin, Sebastian Schwartzkopff, Florian Bertram, Oleg Konovalov, Alexander Hinderhofer, Dmitry Lapkin and Frank Schreiber

Table S1: Performance comparison of Python packages for opening `HDF5` files. The evaluation was performed on a PC [Intel i5-6500, 4 cores, 16 GB RAM, Windows 10, CPU]. The average opening times were calculated over 100 runs.

**Python Package**	**First opening time, ms**	**Averaged time, ms**
PyTables	307.78	26.26 ± 4.73
netCDF4	303.50	23.62 ± 2.05
h5netcdf	608.30	13.87 ± 2.15
H5py	287.97	11.34 ± 2.50

Table S2: Recommendations for metadata fields to be saved with converted data.

**Metadata field**	**Output `HDF5`-file path**	**Required?**	**NXsas**	**Description**
instrument_name	entry/instrument/ name	Yes	Yes	Name of the instrument used to perform the experiment, e.g. 'ID10'
source_type	entry/instrument/ source/type	Yes	Yes	Type of radiation source, e.g. 'synchrotron'
source_probe	entry/instrument/ source/probe	Yes	Yes	Name of radiation probe, e.g. 'x-ray' or 'neutron'
wavelength_spread	entry/instrument/ monochromator/ wavelength_spread	No	Yes	Ratio of wavelength spread to the mean wavelength $\Delta\lambda/\lambda$, used for resolution calculations
start_time	entry/start_time	No	Yes	Measurement start time, e.g. '2021-03-29T15:51:41.343788'
end_time	entry/end_time	No	Yes	Measurement end time, e.g. '2021-03-29T18:45:01.125978'
source_name	entry/instrument/ source/name	No	Yes	Name of the radiation source, e.g. 'ESRF'
filename	entry/data/filename	No	No	Raw pattern file location, e.g. 'data/DIP.h5'
detector_name	entry/instrument/ detector/name	No	No	Name of the detector used, e.g. 'Pilatus300k'
measurement_ technique	entry/instrument/	No	No	experiment geometry, e.g. 'GID' or 'transmission'
measurement_ mode	entry/instrument/	No	No	measurement mode, e.g. 'angular scan'
name	entry/sample/name	Yes	Yes	Descriptive name of the sample, e.g. 'DIP'
preparation	entry/sample/ preparation	No	No	Sample preparation details, e.g. 'gradient thin film prepared by thermal evaporation'
experimental_ conditions	entry/sample/ experimental_ conditions	No	No	Details of the experiment, e.g. 'standard conditions, on air'
structure	entry/sample/ structure structure	Yes	No	Sample structure and materials (Listing S1),

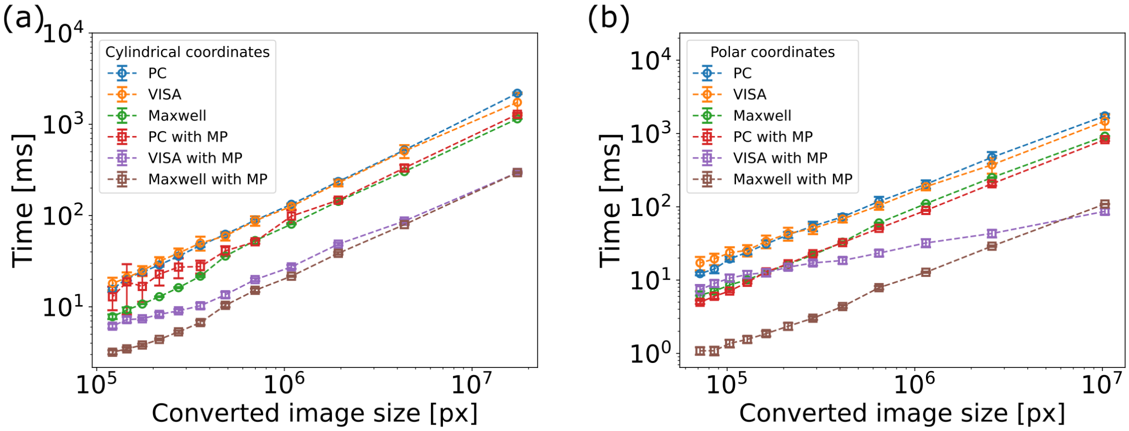

Figure S1: Performance results of the `pygid` package: dependence of coordinate map calculation time on converted image size (resolution) for cylindrical (a) and polar coordinates (b).

A typical GIWAXS pattern was converted to polar coordinates with a resolution of $dq = 0.001$ Å^{-1} and $d\chi = 0.03°$. Simple binning (”NEAREST” interpolation) produces the roughest image (Fig. S2a), whereas for the other interpolation types no significant differences are observed overall. However, variations become more apparent near masked pixels, such as detector gaps and dead pixels. The regions containing masked (NaN) pixels tend to expand more noticeably when using ”CUBIC” and ”LANCZOS4” interpolations, as these methods smooth over larger neighborhoods, whereas NEAREST interpolation shows a local decrease in intensity around masked pixels because missing values are replaced by the nearest valid neighbor without averaging, leading to sharper but less physically accurate transitions. One-dimensional line profiles extracted from the vertical region ($88.5° < \chi < 89°$) reveal intensity fluctuations at small q values (0.15–0.18 Å^{-1}), while the profiles at higher q remain identical (Fig. S2f).

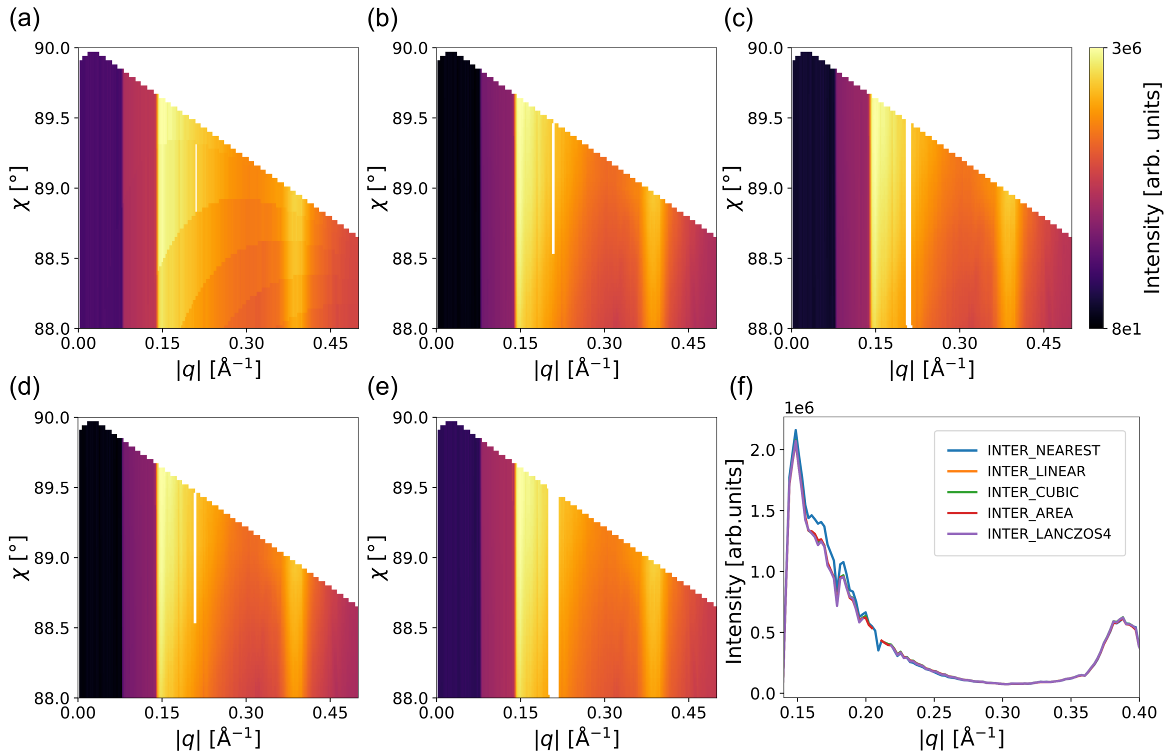

Figure S2: Comparison of interpolation methods used in two-dimensional image conversion: NEAREST (a), LINEAR (b), CUBIC (c), AREA (d), and LANCZOS4 (e). One-dimensional radial profiles are shown for the region near the missing wedge ($88.5° < \chi < 89°$) (f).

Table S3: YAML-formatted metadata for DIP thin film


```yaml
data:
  name: 240306_DIP
  structure:
    stack: air | DIP 0-25e-9| SiOx 1e-9| Si
    materials:
      DIP:
        name: diindenoperylene DIP
        thickness: 25e-9
        cif: DIP.cif
        type: gradient film
        cas_number:     188-94-3
      SiOx:
        name: native SiOx
        thickness: 1e-9
        cas_number:  7631-86-9
      Si:
        name: Si wafer
        cas_number: 7440-21-3
  preparation: gradient thin film prepared by thermal evaporation
  experimental_conditions: standard conditions, on air
```
